# What Stimuli Are Necessary for Anchoring Effects to Occur?

**DOI:** 10.3389/fpsyg.2021.602372

**Published:** 2021-03-12

**Authors:** Yutaro Onuki, Hidehito Honda, Kazuhiro Ueda

**Affiliations:** ^1^Graduate School of Arts and Sciences, The University of Tokyo, Tokyo, Japan; ^2^Japan Society for the Promotion of Science (JSPS), Tokyo, Japan; ^3^Faculty of Psychology, Otemon Gakuin University, Osaka, Japan

**Keywords:** judgment, anchoring effect, numerical priming, semantic priming, scale distortion

## Abstract

The anchoring effect is a form of cognitive bias in which exposure to some piece of information affects its subsequent numerical estimation. Previous studies have discussed which stimuli, such as numbers or semantic priming stimuli, are most likely to induce anchoring effects. However, it has not been determined whether anchoring effects will occur when a number is presented alone or when the semantic priming stimuli have an equivalent dimension between a target and the stimuli without a number. We conducted five experimental studies (*N* = 493) using stimuli to induce anchoring effects. We found that anchoring effects did not occur when a number was presented alone or when phrases to induce semantic priming were used without presenting a number. These results indicate that both numerical and semantic priming stimuli must be presented for anchoring effects to occur. Our findings represent a substantial contribution to the literature on anchoring effects by offering insights into how these effects are generated.

## Introduction

The anchoring effect is a form of cognitive bias in which exposure to some piece of information affects its subsequent numerical estimation (Tversky and Kahneman, 1974). For example, in the study of Tversky and Kahneman (1974), participants first watched a wheel with numbers that stopped either at 65 or 10; they were then asked to perform the following two tasks: (1) a comparison task concerning whether the percentage of African nations in the United Nations was higher or lower than 65 (or 10), and (2) an estimation task concerning the percentage of African nations in the United Nations. In this experimental procedure, when the high anchor (i.e., 65) was presented in the comparison task, the median of numerical estimations in the estimation task was 45%; in contrast, when the low anchor (i.e., 10) was presented in the comparison task, the median of estimations in the estimation task was 25%.

Many researchers have adopted many perspectives to examine how anchoring effects are generated. In this study, we focused on the following two psychological processes: numerical priming and semantic priming. Previous studies have argued that anchoring effects occur by numerical priming (Jacowitz and Kahneman, 1995; Wilson et al., 1996; Wong and Kwong, 2000). For instance, Wong and Kwong (2000) found that the anchor “7,300 m” induced higher numerical estimates than the anchor “7.3 km,” even when the two expressions indicate the same distance. Tversky and Kahneman (1974) concluded that presenting numbers irrelevant to the judgments could generate the anchoring effect. These findings suggest that presenting specific numbers as anchors strongly influences the occurrence of anchoring effects.

Other studies have shown that semantic priming can induce anchoring effects (Strack and Mussweiler, 1997; Mussweiler and Strack, 1999a,b, 2001; Mussweiler et al., 2000). Strack and Mussweiler (1997) found that, when participants were first asked whether the Brandenburg Gate was higher or lower than 150 m (or 25 m) and were then asked about its height, the anchor value significantly affected their estimations of the Brandenburg Gate’s height. However, when they were first asked whether the Brandenburg Gate was wider or narrower than 150 m (or 25 m) and were then asked about its height, the anchor value did not affect their subsequent estimations of the height of the Brandenburg Gate. These findings suggest that, when the dimension of an anchor value (e.g., width) does not correspond with the dimension of a subsequent estimation (i.e., height), the anchor value does not affect the subsequent estimation. This suggests that the anchoring effect can be explained by the semantic priming model. When an anchor represents a height dimension, this activates knowledge related to the height dimension that is relevant to subsequent numerical estimations of height. Therefore, in this case, the anchoring effect is generated. In contrast, when an anchor represents a width dimension, this activates knowledge related to the width dimension that is irrelevant to subsequent estimations of height, so the anchoring effect is not generated. In other words, when the dimension of an anchor value is the same as that used in a subsequent estimation, the anchor affects the numerical estimation. According to the semantic priming model, the occurrence of anchoring effects requires stimuli that represent either high or low anchor values (semantic information) as well as an equivalent dimension between the stimuli and the target. Therefore, for this study, we designed our semantic priming stimuli as stimuli that combined semantic information with dimension information.

The numerical and semantic priming models are two possible candidates for explaining the generation of anchoring effects. However, in the strict sense, the anchor values used in many previous studies were neither numerical nor semantic priming stimuli. Moreover, conventional experimental procedures on anchoring effects involve two tasks: a comparison task (presenting an anchor value) and a subsequent numerical estimation task. In its task nature, semantic priming stimuli should be given to the number presented in the comparison task. For example, if the study participants are asked to compare the percentage of African nations in the United Nations with 65, as in the study of Tversky and Kahneman (1974), they might use 65% (i.e., a stimulus combining numerical information with semantic information) by spontaneously adding units to 65. That is, in comparison tasks, even if only numbers are used, participants might add units to the numbers. Therefore, even when only numbers are used as stimuli, we still do not know whether or not numbers alone might produce the anchoring effect or whether presenting both a number and semantic priming stimuli might generate the anchoring effect.

To disentangle the issue above, we examined what stimuli were necessary to produce anchoring effects. The subsequent sections are organized as follows: in experiments 1 and 2, we strictly controlled the anchor value as a “numerical” or a “semantic” priming stimulus and examined whether such anchors generated anchoring effects. Based on the findings from those experiments, we proposed a new hypothesis about the features of anchors that would produce anchoring effects; then, we tested this hypothesis in experiment 3. The specific experimental methods are described in detail in the respective experimental sections.

## Experimental Study

The protocols of the following experiments conformed to the Declaration of Helsinki and were approved by the Ethics Review Committee for Experimental Research with Human Subjects at the University of Tokyo.

### Experiment 1a

We examined whether the presentation of a number alone would generate the anchoring effect. As mentioned in the introduction, using a comparison task makes it difficult to use a number alone as a stimulus. Thus, in experiment 1, we followed a new experimental procedure that used only a number as an anchor and examined whether the anchor affected the subsequent numerical estimation task. In accordance with the numerical priming model, we expected this anchor to affect the subsequent estimation.

### Experiment 1a Participants

We recruited a total of 60 participants (*M*_age_ = 46.98, *SD*_age_ = 11.19, women = 33, men = 27) from Rakuten Insight^1^, a market research firm in Japan^2^. We randomly assigned them to one of two groups: the high anchor group (*n* = 29) and the low anchor group (*n* = 31). We used the program G^∗^Power, version 3.1.9.3 (Faul et al., 2007), to compute the required sample size. In Guthrie and Orr’s (2006) meta-analysis of anchoring effects, the smallest effect size of anchoring was *r* = 0.30. Thus, we set the effect size to *r* = 0.30 for the sample size analysis; when we used the program G^∗^Power, we used an effect size converter^3^ to convert the effect size *r* = 0.30 to *d* = 0.629. Moreover, we used a one-tailed analysis because we hypothesized that a high-value estimation would be more likely in the high anchor group than in the low anchor group. The analysis indicated that a sample size of approximately 30 participants would be necessary for the study to have a detection power of 80% and α = 0.05. Thus, we tried to recruit at least 30 participants for each group^4^.

#### Experiment 1a Task, Stimulus, and Procedure^5^

**FIGURE 1:**
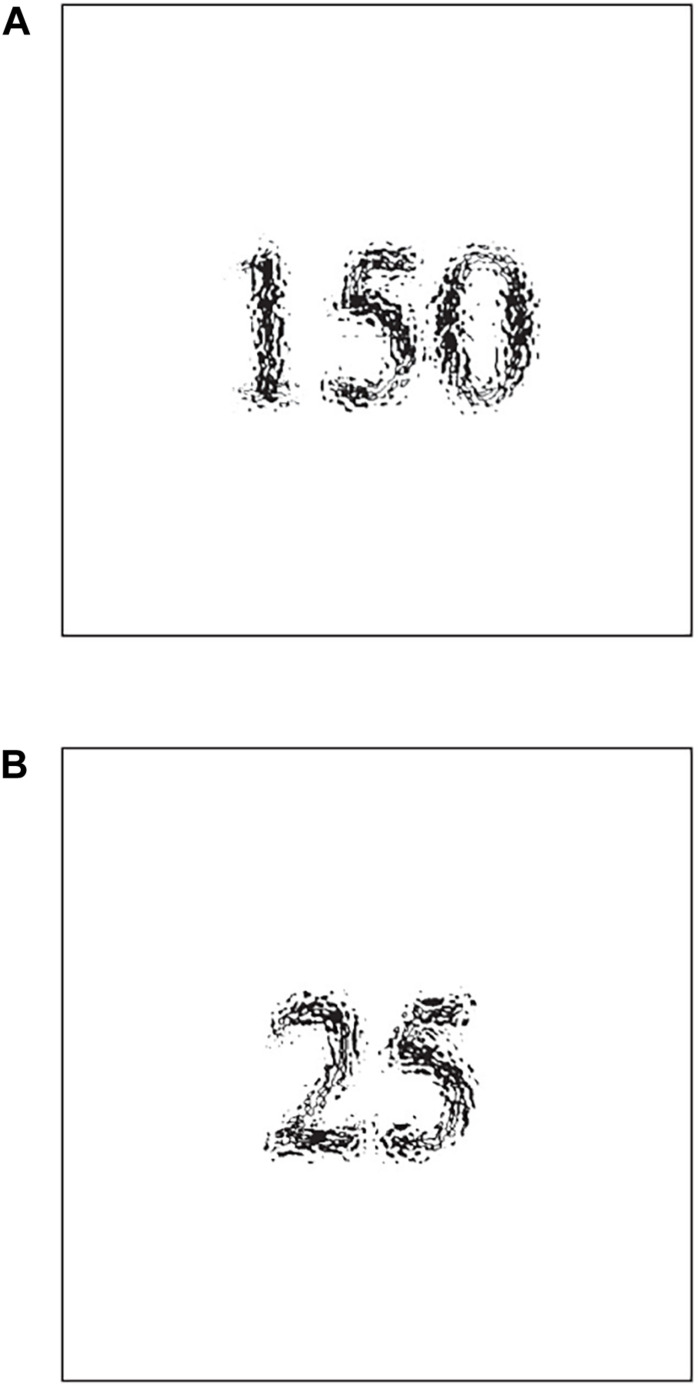
**(A)** Stimulus for the high anchor group (“150”). **(B)** Stimulus for the low anchor group (“25”).

We conducted this experiment using the GUI in Qualtrics^6^^,7^. We first presented the participants with a number on their computer screens and asked what the number represented. The number continued to be presented until the participants answered. Figure 1 shows the numbers we presented to the high (150; Figure 1A) and low anchor (25; Figure 1B) groups. After they answered that question, we asked the participants, “Could you estimate the average weight of Czechs?” and “Do you know the correct answer about the average weight of Czechs?” In all tasks, there was no time limit on the response time.

We used “the average weight of Czechs” as the target as we assumed that most Japanese people would not know the correct answer, but would be able to arrive at adequate estimates. Moreover, following the experimental procedure of Strack and Mussweiler (1997), we used 150 and 25 as our anchors. When disfluent material (i.e., different fonts) was presented, the participants recalled the material more than when fluent material was presented since they adopted deeper processing strategies (Diemand-Yauman et al., 2011). We expected that as the cognitive processing of the stimuli became deeper, the impact of the stimuli on the anchoring effect would increase. For this reason, we used hard-to-read fonts (Figure 1, blurred text in black). The font manipulation (e.g., a small, gray, italicized, or condensed font) could produce disfluency (Alter and Oppenheimer, 2009; Diemand-Yauman et al., 2011). Diemand-Yauman et al. (2011) used slightly lighter colors of letters as disfluent characters. Therefore, we defined and used the stimuli as hard-to-read fonts (Figure 1)^8^.

#### Experiment 1a Results

We excluded one datum in the low anchor group because the participant knew the correct answer in advance.

**FIGURE 2:**
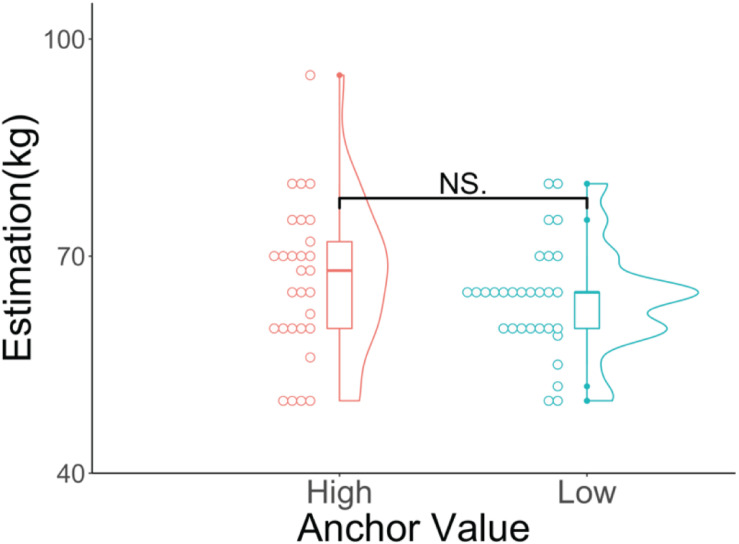
Violin and dot plots of the participants’ estimations. The *y*-axis shows the estimations for the average weight of Czechs (in kilograms). ^NS^*p* > 0.05.

Figure 2 shows the results of the participants’ estimations of the average weight of Czechs for the two groups. The Wilcoxon rank-sum test showed that there was no significant difference between the high (median = 68, *SD* = 10.53, mean rank = 32.50) and low anchor (median = 65, *SD* = 7.49, mean rank = 27.58; *p* = 0.265, *z* = 1.11, *r* = 0.15) groups.

#### Experiment 1a Discussion

We found that the presentation of a number alone did not yield anchoring effects. This result was not predicted by the numerical priming model.

### Experiment 1b

The results obtained in experiment 1a were different from what the numerical priming model predicted. Therefore, we conducted experiment 1b to see whether we could replicate the results of experiment 1a with different participants to increase the reliability of the experimental results.

As shown in the previous sample size analysis, we tried to recruit at least 30 participants per group for the anchoring experiment. However, there were 29 participants in the high anchor group in experiment 1a, so the number of participants in that high anchor group was smaller than the required number. Therefore, we ensured that the number of participants in experiment 1b was higher than the number in experiment 1a.

### Experiment 1b Participants

We recruited a total of 64 participants (*M*_age_ = 47.04, *SD*_age_ = 8.52, women = 29, men = 35) from Rakuten Insight and randomly assigned them to one of the two groups: the high anchor group (*n* = 33) and the low anchor group (*n* = 31).

#### Experiment 1b Task, Stimulus, and Procedure

The experimental stimuli and tasks were the same as in experiment 1a.

#### Experiment 1b Results

We excluded one datum from the low anchor group because the answer was an outlier (the estimation of the average weight of Czechs was 1 kg). The Smirnov–Grubbs test showed that the lowest value of 1 kg was an outlier (*p* < 0.001).

**FIGURE 3:**
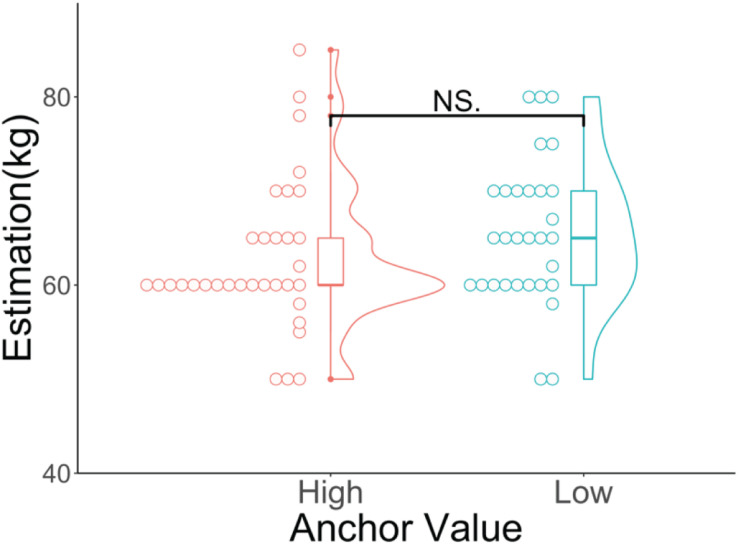
Violin and dot plots of the participants’ estimations. The *y*-axis shows the estimations for the average weight of Czechs (in kilograms). ^NS^*p* > 0.05.

Figure 3 shows the results of the participants’ estimations of the average weight of Czechs for the two groups. The Wilcoxon rank-sum test showed that there was no significant difference between the high anchor (median = 60, *SD* = 7.93, mean rank = 28.22) and low anchor (median = 65, *SD* = 7.71, mean rank = 36.15; *p* = 0.079, *z* = −1.76, *r* = 0.22) groups.

#### Experiment 1b Discussion

In experiment 1b, we obtained a result similar to that in experiment 1a. Thus, presenting only a number did not produce anchoring effects.

### Experiment 2a

The results of experiments 1a and 1b showed that the anchoring effect did not occur when we presented a number alone. As mentioned in the introduction, the numerical and semantic priming models are two possible candidates for explaining the generation of anchoring effects. In experiment 2a, we created a new experimental stimulus that used only semantic priming stimuli. In particular, we examined whether the anchoring effects would occur when we presented semantic priming stimuli without numbers (e.g., “a very heavy person” or “a very light person”). According to the semantic priming model, it is necessary to indicate either a high or low anchor value and to align the dimension between a stimulus and a target to generate the anchoring effect. For this reason, we used stimuli that showed both semantic (i.e., high or low) and dimensional (i.e., weight) information. According to the model of semantic priming, these anchors could affect subsequent numerical estimations.

### Experiment 2a Participants

We recruited a total of 58 participants (*M*_age_ = 44.84, *SD*_age_ = 11.04, women = 39, men = 19) and randomly assigned them to one of two groups: the high anchor value group (*n* = 29) and the low anchor value group (*n* = 29).

#### Experiment 2a Task, Stimulus, and Procedure

We first asked the participants whether the average weight of Czechs was heavier or lighter than “a very heavy person” (high anchor) or “a very light person” (low anchor). The anchor continued to be presented until the participants answered. They were then asked to estimate the average weight of Czechs and to indicate whether they knew the correct answer about the average weight of Czechs. In all tasks, there was no time limit on the response time.

#### Experiment 2a Results

We excluded the data of two participants (one from the high group and the other from the low anchor group) because they indicated that they knew the correct answer.

**FIGURE 4:**
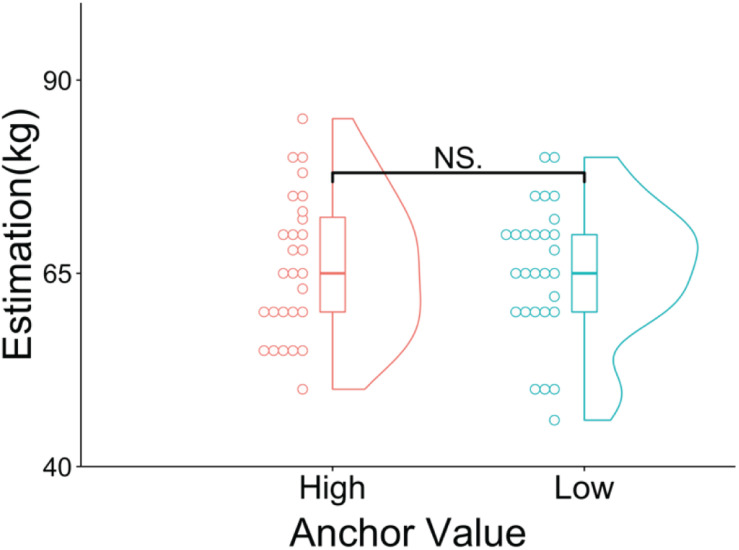
Violin and dot plots of the participants’ estimations. The *y*-axis shows the estimations for the average weight of Czechs (in kilograms). ^NS^*p* > 0.05.

Figure 4 shows the results of the participants’ estimations of the average weight of Czechs for the two groups. The Wilcoxon rank-sum test showed that there was no significant difference between the high (median = 65, *SD* = 9.13, mean rank = 28.54) and low anchor (median = 65, *SD* = 8.87, mean rank = 28.46; *p* = 0.987, *z* = 0.017, *r* = 0.002) groups.

#### Experiment 2a Discussion

We found that anchoring effects did not occur only when we presented phrases that we expected to induce semantic priming. This result was not predicted by the semantic priming model.

### Experiment 2b

In experiment 2a, no anchoring effect occurred with the semantic priming stimuli, contrary to what the semantic priming model predicted. Therefore, we conducted experiment 2b to see whether we could replicate the results of experiment 2a with different participants to increase the reliability of the experimental result.

In experiment 2a, there were 29 participants in both high and low anchor groups, so the number of participants in both groups did not reach the required number. Therefore, we ensured that the number of participants in experiment 2b was greater than that in experiment 2a.

#### Experiment 2b Participants

We recruited a total of 69 participants (*M*_age_ = 43.69, *SD*_age_ = 9.23, women = 47, men = 22) and randomly assigned them to one of two groups: the high anchor value group (*n* = 36) or the low anchor value group (*n* = 33).

#### Experiment 2b Task, Stimulus, and Procedure

The experimental stimuli and tasks were the same as those in experiment 2a.

#### Experiment 2b Results

We excluded the datum for one participant (low anchor) who knew the correct estimation answer.

**FIGURE 5:**
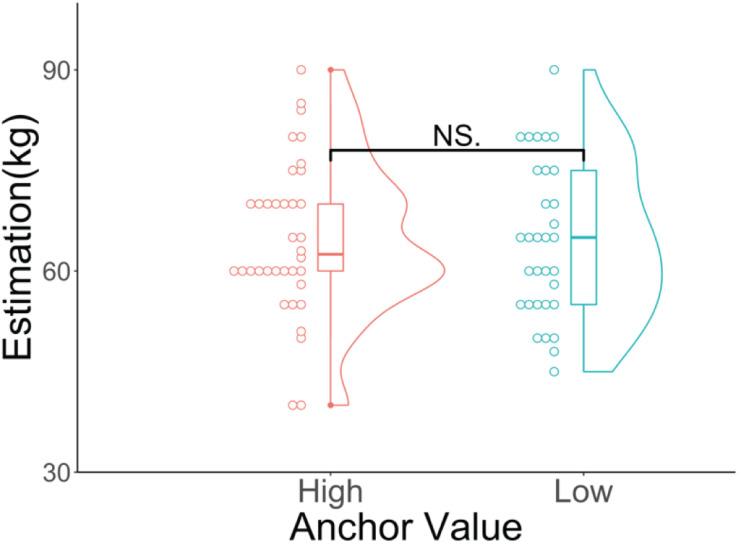
Violin and dot plots of the participants’ estimations. The *y*-axis shows the estimations for the average weight of Czechs (in kilograms). ^NS^*p* > 0.05.

Figure 5 shows the results of the participants’ estimations of the average weight of Czechs for the two groups. The Wilcoxon rank-sum test showed that there was no significant difference between the high (median = 62.5, *SD* = 11.45, mean rank = 35.25) and low anchor groups (median = 65, *SD* = 11.51, mean rank = 33.65; *p* = 0.738, *z* = 0.334, *r* = 0.04).

#### Experiment 2b Discussion

We found that no anchoring effects occurred when we presented only phrases that we expected to induce semantic priming. These results were similar to those obtained in experiment 2a.

### Experiment 3

The results of experiments 1 and 2 showed that no anchoring effects occurred when we presented only a number or when we presented phrases intended to induce semantic priming, but included no number. Previous studies on the anchoring effect have used numerical and semantic priming stimuli simultaneously. Considering this, along with the findings from our experiments 1 and 2, we proposed a new hypothesis concerning the anchoring effect: generating anchoring effects requires the simultaneous presentation of numerical and semantic priming stimuli. Thus, we then set out to examine whether we could induce the anchoring effect by using both numerical and semantic priming stimuli at the same time. We believe that the synergistic effect or the interaction between the numerical and semantic priming stimuli is likely to induce the anchoring effect. For this reason, in experiment 3, we tested the hypothesis that the interaction between the numerical and semantic priming stimuli would induce the anchoring effect.

To examine the interaction, we created a new stimulus to directly examine how the presence or absence of numerical and semantic priming stimuli would affect the occurrence of the anchoring effect. Specifically, we used a two (presenting numerical priming stimuli or not) × two (presenting semantic priming stimuli or not) factorial design. This established four groups: the first group (hereinafter referred to as the NumSem group) was presented with numerical and semantic priming stimuli [e.g., “a very heavy person 150 (kg)”]; the second group (hereinafter referred to as Num group) was presented with stimuli with only a number (e.g., 150); the third group (hereinafter referred to as the Sem group) was presented with the semantic priming stimuli without a number [e.g., “a very heavy person (kg)”]; and the final group (hereinafter referred to as the Control group) was presented with stimuli that had neither numerical nor semantic priming stimuli (i.e., we did not show any anchor to participants). Except for the Control group, we established two groups (high and low anchor values) for each of the NumSem, Num, and Sem groups.

The anchoring effect is usually investigated by the comparison of numerical estimations between the high and low anchor groups (e.g., Tversky and Kahneman, 1974). However, there is another commonly used method to investigate the anchoring effect: comparing the estimated values between the control group and the anchor presented group—that is, comparing one group that has been presented with either a high or a low anchor with a control group (e.g., Frederick and Mochon, 2012; Harris and Speekenbrink, 2016). Since the purpose of experiment 3 was to examine whether the interaction between the numerical and semantic priming stimuli would occur, we needed to have a control group to whom no anchor was presented. Thus, we compared the estimates of the Control group with those of the groups presented with high anchor values and low anchor values separately. Specifically, we performed a two (presenting numerical priming stimuli or not) × two (presenting semantic priming stimuli or not) ANOVA for each anchor value. We note that the data of the Control group for analysis was the same between the high and low anchor conditions.

#### Experiment 3 Participants

We recruited a total of 242 participants (*M*_age_ = 45.35, *SD*_age_ = 11.32, women = 108, men = 131, those who responded that they did not want to identify their gender = 3) and randomly assigned them to one of the seven groups (see Table 1).

**TABLE 1:** Stimuli used in experiment 3.

	Numerical priming stimuli	Semantic priming stimuli	Value	*n*
150	(+)	(−)	High	33
25	(+)	(−)	Low	34
A very heavy person (kg)	(−)	(+)	High	33
A very light person (kg)	(−)	(+)	Low	36
A very heavy person 150 (kg)	(+)	(+)	High	37
A very light person 25 (kg)	(+)	(+)	Low	37
Control	(−)	(−)	None	32

*The numerical priming stimuli column shows whether the anchors included a number or not; the semantic priming stimuli column shows whether the anchors included semantic priming stimuli or not (for both, + = yes, − = no). Value column shows whether the anchor value was high, low, or none.*

#### Experiment 3 Task, Stimulus, and Procedure

**FIGURE 6:**
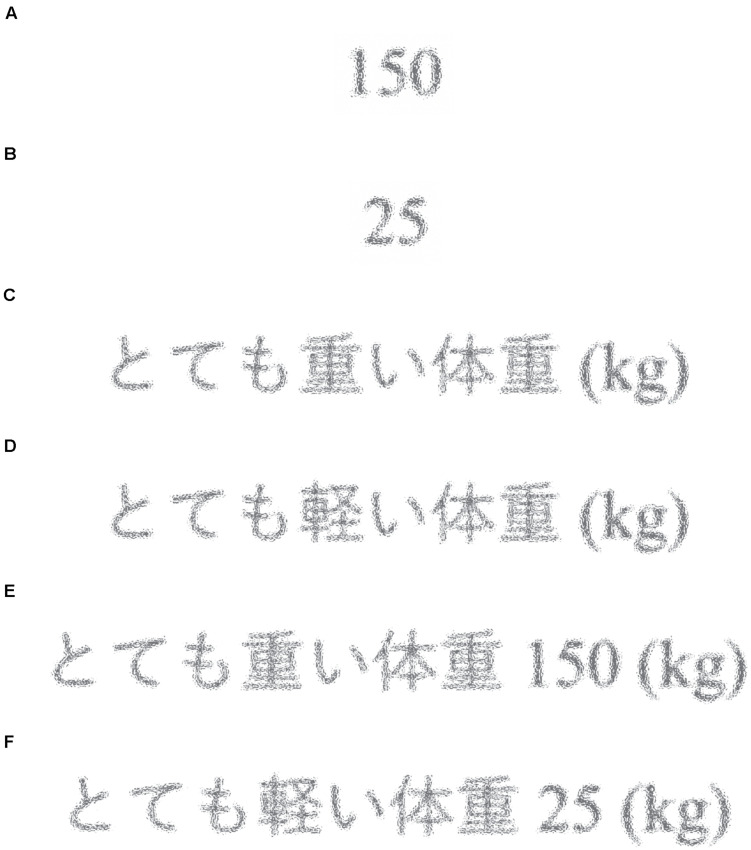
**(A)** Stimulus for the high anchor Num group (“150”). **(B)** Stimulus for the low anchor Num group (“25”). **(C)** Stimulus for the high anchor Sem group [“a very heavy person (kg)”]; the original was written in Japanese. **(D)** Stimulus for the low anchor Sem group [“a very light person (kg)”]; the original was written in Japanese. **(E)** Stimulus for the high anchor NumSem group [“a very heavy person 150 (kg)”]; the original was written in Japanese. **(F)** Stimulus for the low anchor NumSem group [“a very light person 25 (kg)”]; the original was written in Japanese.

In all groups, the target of estimation was “the average weight of Czech men.” As with experiments 1a and 1b, we presented the participants with an anchor (see Figure 6) and asked them what the stimuli represented. The anchor continued to be presented until the participants answered. Subsequently, we asked the participants to estimate the number of the target. Finally, we asked whether they knew the correct answer. For the Control group, neither numbers nor semantic priming stimuli were presented. In all tasks, there was no time limit on the response time.

#### Experiment 3 Results

We excluded the data for 11 participants: one low Num group participant knew the correct answer, nine participants did not correctly recognize the hard-to-read characters (one from the low Num group, two from the low Sem group, three from the high Sem group, two from the low NumSem group, and one from the high NumSem group), and one participant in the low Sem group had an outlier answer (the estimation of the average weight of Czech men was 6 kg). The Smirnov–Grubbs test showed that the lowest value of 6 was an outlier (*p* < 0.001).

**FIGURE 7:**
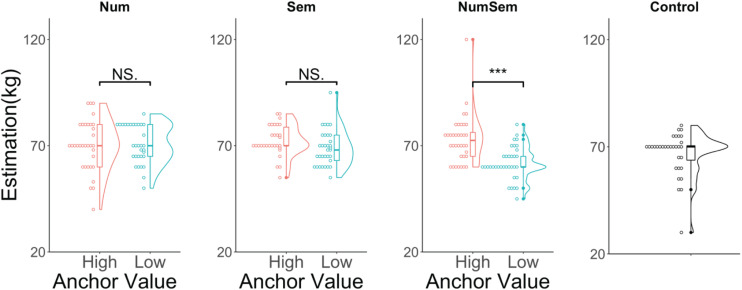
Violin and dot plots of the participants’ estimations. The *y*-axis shows the estimations for the average weight of Czech men (in kilograms). ***p < 0.001, ^NS^*p* > 0.05.

We examined whether each corresponding anchor between the high and low anchor groups produced anchoring effects (see Figure 7). The multiple comparisons using the Wilcoxon rank-sum test with a Bonferroni correction revealed no significant difference between “150” (Num high group; median = 70, *SD* = 12.03, mean rank = 33.02) and “25” (Num low group; median = 70, *SD* = 8.98, mean rank = 32.98, *p* = 0.999, *z* = 0.007, *r* = 0.001). Similarly, there was no significant difference between “a very heavy person (kg)” (Sem high group; median = 70, *SD* = 7.20, mean rank = 37.32) and “a very light person (kg)” (Sem low group; median = 68, *SD* = 8.31, mean rank = 27.17, *p* = 0.079, *z* = 2.22, *r* = 0.28). However, there was a significant difference between “a very heavy person 150 (kg)” (NumSem high group; median = 72.5, *SD* = 11.39, mean rank = 46.76) and “a very light person 25 (kg)” (NumSem low group; median = 60, *SD* = 7.73, mean rank = 24.93, *p* < 0.001, *z* = 4.53, *r* = 0.54).

**FIGURE 8:**
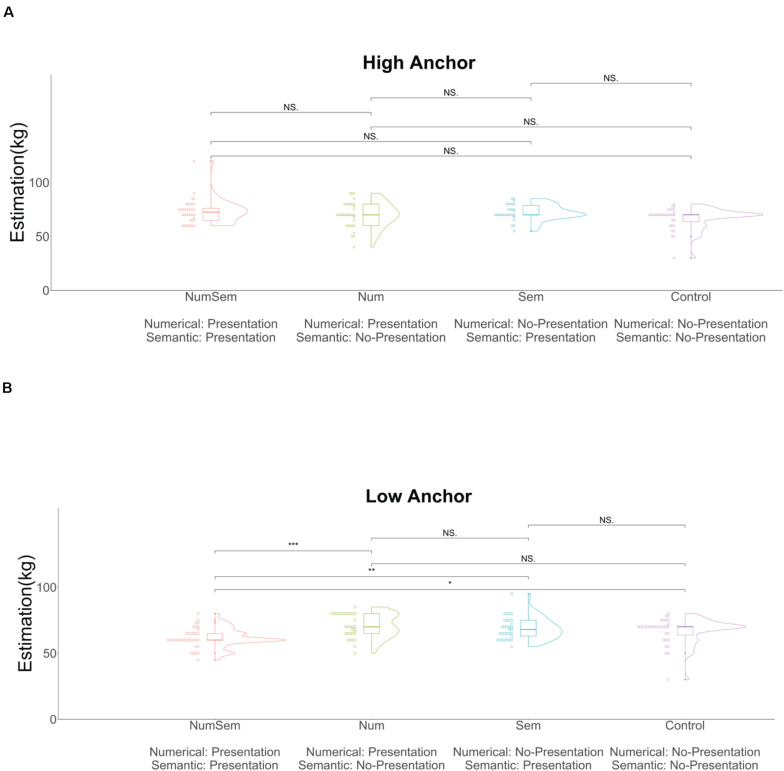
Violin and dot plots of the participants’ estimations. **(A)** Data for the high anchor value. **(B)** Data for the low anchor value. The *y*-axis shows the estimations for the average weight of Czech men (in kilograms). The data of the Control group were the same between the high and low anchor conditions. “Numerical” = whether the anchor contained numerical priming stimuli or not; “Semantic” = whether the anchor contained semantic priming stimuli or not. ***p < 0.001, **p < 0.01, *p < 0.05, ^NS^*p* > 0.05.

We conducted a two (presentation of numerical priming stimuli or not) × two (presentation of semantic priming stimuli or not) ANOVA on the estimation. We conducted this analysis for each anchor value. That is, we analyzed the data for (the presentation of numerical priming stimuli of high anchor value or not) × (the presentation of semantic priming stimuli of high anchor value or not) and for (the presentation of numerical priming stimuli of low anchor value or not) × (the presentation of semantic priming stimuli of low anchor value or not). The analysis for the high anchor values (Figure 8A) indicated no significant main effect of the numerical priming stimuli [*F*(1, 127) = 1.27, *p* = 0.262, η^2^ = 0.009] and no significant interaction between them [*F*(1, 127) = 0.58, *p* = 0.445, η^2^ = 0.004]. However, it did find a significant main effect for the semantic priming stimuli [*F*(1, 127) = 5.22, *p* = 0.024, η^2^ = 0.039]. The analysis for the low anchor values (Figure 8B) also showed no significant main effect of the numerical priming stimuli [*F*(1, 128) = 1.28, *p* = 0.259, η^2^ = 0.009]. However, it did find a significant main effect for the semantic priming stimuli [*F*(1, 128) = 5.29, *p* = 0.022, η^2^ = 0.036] and a significant interaction between them [*F*(1, 128) = 12.50, *p* < 0.001, η^2^ = 0.085]. We performed multiple comparisons by using the Wilcoxon rank-sum test with a Bonferroni correction on the data for the high anchor values and found no significant differences between any pairs of groups. Similarly, we performed multiple comparisons on the data for the low anchor values and found the following results. The estimated value in the “a very light person 25 (kg)” (NumSem group; median = 60, *SD* = 7.73, mean rank = 25.36) group was significantly lower than that in the “25” (Num group; median = 70, SD = 8.98, mean rank = 43.45, *p* < 0.001, *z* = −3.86, *r* = 0.47) group. The estimated value in the “a very light person 25 (kg)” (NumSem group; median = 60, *SD* = 7.73, mean rank = 27.07) group was significantly lower than that in the “a very light person (kg)” (Sem group; median = 68, *SD* = 8.31, mean rank = 42.38, *p* = 0.007, *z* = −3.25, *r* = 0.39) group. The estimated value in the “a very light person 25 (kg)” (NumSem group; median = 60, *SD* = 7.73, mean rank = 27.47) group was significantly lower than that in the Control group (median = 70, *SD* = 10.17, mean rank = 41.14, *p* = 0.021, *z* = −2.92, *r* = 0.36). We found no significant differences between any other group pairs.

#### Experiment 3 Discussion

We examined whether each of the anchors between the high and low anchor groups produced anchoring effects and found that anchoring effects only occurred when we presented numerical and semantic priming stimuli simultaneously. However, no anchoring effects occurred when we presented numerical priming stimuli or semantic priming stimuli alone. These results show that the simultaneous presentation of numerical and semantic priming stimuli is necessary to generate anchoring effects. Moreover, we compared the Control group estimates with those of each anchor group separately for the high and low anchor values. We found a significant interaction between the low anchor group’s numerical and semantic priming stimuli. The effect of anchors is stronger with low anchors than with high anchors (Hardt and Pohl, 2003). This is probably why we observed a significant interaction only for the low anchor values. Our results demonstrated that the influences of numerical and semantic priming stimuli on the anchoring effect were interdependent. The combined results mean that the interaction between the numerical and semantic priming stimuli generates the anchoring effect.

## General Discussion

In this study, we conducted five experiments to examine whether anchoring effects occurred when we presented a number alone or phrases that induced semantic priming without using a number. The results showed that no anchoring effects occurred with only a numerical or a semantic priming stimulus. However, when we presented both numerical and semantic priming stimuli simultaneously, anchoring effects did occur. Thus, our experiments indicated that to generate anchoring effects requires the presence of both numerical and semantic priming stimuli. These results suggest that while numerical priming stimuli and semantic priming stimuli alone have no or very weak anchor effects on subsequent estimations, they have a synergistic effect when combined that increases the likelihood of anchoring effects.

Previous research has established that the degree of compatibility between the input and the output stimulus attributes affects judgment and decision making. When the presented stimulus is highly compatible with the subsequent response, the response is more strongly influenced, as expressed in the compatibility principle (Tversky et al., 1988; Slovic et al., 1990). For example, in wagers, given a choice between a high probability of winning a small amount of money (e.g., 35/36 chances to win $4) and a low probability of winning a large amount of money (e.g., 11/36 chances to win $16), more people would choose the former; however, asked about the lowest selling price for each bet, about half stated prices that are inconsistent with their choices (Slovic et al., 1990). People tend to be affected more strongly by the amount of money they can get in the wager than by the probability of winning because the response (e.g., the lowest selling price people can pay) is asked with the dimension of money. In the current experiments, an anchor needed numerical and semantic priming stimuli to cause the anchor effect. According to studies on the compatibility principle, when an anchor contains not only a number but also semantic information consistent with a target dimension, the anchor value is highly compatible with the target value, and this affects the numerical estimation of the target. Thus, our findings are consistent with the compatibility principle.

In experiments 1 and 3, we used “150” and “25” as stimuli, following the experiment of Strack and Mussweiler (1997). However, those numbers have a different number of digits. Wong and Kwong (2000) showed that the anchoring effect changed when different digits with the same meaning were used. In future studies, the effects of numerical and semantic priming stimuli should be examined by more rigorously controlling the number of digits.

In addition, we conducted a within-participants experiment using the same stimuli in experiment 3, except for the Control group. That is, we examined whether each anchor between the high and low anchor conditions produced anchoring effects in a within-participants experimental design. The experimental procedure differed from that in experiment 3. We recruited a total of 168 participants (*M*_age_ = 44.47, *SD*_age_ = 11.51, women = 80, men = 87, those who responded that they did not want to identify their gender = 1) from Rakuten Insight. We first presented the participants with one of the anchors (see Figure 6) and asked what the stimulus represented. The anchor continued to be presented until the participants answered. Then, we asked them to estimate the number of the target (the average weight of Czech men), followed by the instruction: “In the previous session, you answered the question. In the following question, you will be asked to answer the same question again. You are allowed, but not obliged, to change your mind and provide an amended answer.” After this instruction, they were presented with another anchor, asked what the stimulus represented, and asked to predict the average weight of Czech men, and so on. In all tasks, there was no time limit on the response time. Note that we presented different values (low and high anchor values) in the same anchor group to the same participants. For example, we first presented “150” to the Num group, then presented “25” as the next stimulus. We presented the high and low values in random order. The above experimental procedure was basically the same as that used by Teovanović (2019). We found no significant difference between any of the high and low anchor values. In contrast, Teovanović’s (2019) participants amended their initial estimates toward an anchor in more than half of the cases. In our study, far fewer than half (about 20%) did so. Teovanović (2019) did not use anchors in the first estimation, but we used anchors twice in the experiment; this might be why we obtained different results. As with Tversky and Kahneman’s (1974) study, research on anchoring effects is commonly conducted using a between-participants experimental design. The anchoring effect might be less observable in a within-participants experimental design.

Finally, we must discuss the related psychological mechanism of anchoring effects. Frederick and Mochon (2012) proposed a new idea, the scale distortion theory of anchoring effects. The scale distortion theory holds that an anchor does not change respondents’ internal representations of a target for estimation (e.g., the average weight of Czechs is heavy) but the *scale* for the estimation (Frederick and Mochon, 2012). For example, when respondents estimate a raccoon’s weight in pounds (i.e., anchor value) and then estimate a giraffe’s weight using the same scale, their weight estimate for a raccoon affects their estimate of the giraffe’s weight. When respondents estimate a raccoon’s weight on a seven-point scale (i.e., anchor value) and then estimate a giraffe’s weight in pounds, their estimate of a raccoon’s weight does not affect their estimate of a giraffe’s weight. If the anchoring effects are induced through semantic priming, the anchoring effect occurs regardless of the estimation scale used for the raccoon. Based on these findings, Frederick and Mochon (2012) claimed that “the anchor changes how the response scale is used, not how the focal stimulus is perceived.” That is, anchoring effects can be explained by the distortion of a specific response scale (Frederick and Mochon, 2012). The scale distortion theory states that the scale relationship between an anchor value and a target value is critical for the generation of anchoring effects—that is, that the difference or similarity in scale between the anchor value and the target value affects the likelihood of the occurrence of anchoring effects. The present study discussed the features of an anchor value that can generate anchoring effects and found that both number and semantic priming stimuli are necessary to induce anchoring effects. In our study, the semantic scale of the anchor was the same as that for the target. Thus, the response scale was *not distorted*, so our findings were consistent with those obtained in the scale distortion theory studies.

In sum, the present study showed that anchoring effects did not occur when numerical and semantic priming stimuli without a number were presented alone. In addition, this study showed that both numerical and semantic priming stimuli were needed to generate anchoring effects. Thus, we conclude that a combination of numerical and semantic priming for anchoring effects is needed to understand the mechanism for their occurrence.

## Data Availability Statement

All data and materials (i.e., from experiments 1a, 1b, 2a, 2b, and 3, the within-participants design experiment, and the experiment to measure the legibility of texts) are publicly available via the Open Science Framework and can be accessed at https://osf.io/ynxc7/?view_only=d3dee8120ee14793851a6db6545a3af7.

## Ethics Statement

The studies involving human participants were reviewed and approved by the Ethics Review Committee for Experimental Research with Human Subjects at the University of Tokyo. The patients/participants provided their written informed consent to participate in this study.

## Author Contributions

HH and YO led the data collection for all the experiments. YO led the research and data collection, along with data analyses for all the experiments, and drafted the manuscript. KU and HH provided critical revisions. All authors approved the final version of the manuscript for submission and contributed to the experiment concept and design.

## Conflict of Interest

The authors declare that the research was conducted in the absence of any commercial or financial relationships that could be construed as a potential conflict of interest.

## Footnotes

1 https://insight.rakuten.co.jp/en/

2 Participants in experiments 1a, 1b, 2a, 2b, and 3 were not overlapping.

3 http://escal.site

4 In experiments 1b, 2a, 2b, and 3, the recruiting methods and sample size analyses were the same as in experiment 1a.

5 All experiments were conducted in Japanese with Japanese participants. For this paper, the experimental stimuli have been translated into English.

6 The same GUI was used in experiments 1b, 2a, 2b, and 3, a within-participants design experiment in the General Discussion section and an experiment to measure the legibility of the sample text in the Supplementary Material.

7 http://www.qualtrics.com

8 We conducted an experiment to determine whether differences in fonts affected the legibility of texts. The details of this experiment are described in Supplementary Material.
