# Supplementary material for: What Stimuli Are Necessary for Anchoring Effects to Occur?
Source: Front Psychol. 2021 Mar 12;12:602372. doi: 10.3389/fpsyg.2021.602372 (PMC8006283; doi:10.3389/fpsyg.2021.602372)
Supplement: Supplementary file 1 [file Data_Sheet_1.pdf]

Supplementary Material

1 Supplementary Experiment

People recall hard-to-read fonts more readily than easy-to-read fonts and adopt deeper processing strategies for the former than for the latter (Diemand-Yauman et al., 2011). As the cognitive processing of the stimuli (i.e., anchors) became deeper because the fonts were harder to read, we expected the impact of the stimuli on the anchoring effect to increase. For this reason, in Experiments 1 and 3, we used the hard-to-read fonts as stimuli. However, we could not determine whether the differences in the fonts, such as the stimuli used in the current experiments (i.e., the blurred texts), affected the legibility of stimuli. Thus, we conducted an experiment to elucidate whether differences in the fonts affected the legibility of texts.

2 Participants

We recruited 30 participants (M_{age} = 48.83, SD_{age} = 12.76, women = 10, men = 20, within-participant design) from Rakuten Insight[1], a market research firm in Japan.

3 Task, Stimulus, and Procedure

We used 18 types of stimuli (six types of anchors × three types of fonts; see Figure R1 in the supplementary material). We used these six types of anchors: "150," "25," "a very heavy person (kg)," "a very light person (kg)," "a very heavy person 150 (kg)," "a very light person 25 (kg)." We used these three types of fonts: easy-to-read fonts (i.e., text without any editing), hard-to-read fonts (blurred black text, which were used in Experiment 1), and hard-to-read fonts (blurred gray text, which were used in Experiment 3). The participants were presented with one of the 18 types of stimuli and asked, "Can you read the text?"; they were instructed to choose one of two options: "I cannot read the text" or "I can read the text." In this question, we also measured their response time for selecting the options. After completing this task, they were asked, "How difficult was it for you to read?"; they were instructed to answer on a scale ranging from "I cannot read the text very well" (far left) to "I can read the text very well" (far right), with 101 total points. They answered the two questions for each of the 18 types of stimuli for a total of 36 answers. The 18 types of stimuli were presented in random order.

4 Results and Discussion

We performed multiple comparisons of the Wilcoxon signed-rank test using a Bonferroni correction on the data derived from the answers describing the anchor type's legibility for the three fonts (see Figure R2). The results showed that the easy-to-read fonts were rated significantly easier to read than the hard-to-read fonts (in black). Similarly, the easy-to-read fonts were rated significantly easier to read than the hard-to-read fonts (in gray). However, there were no significant differences in the

---

[1] https://insight.rakuten.co.jp/en/

legibility between the hard-to-read fonts (in black) and the hard-to-read fonts (in gray). The results showed that the hard-to-read fonts (in black or gray) were more difficult to read than the easy-to-read fonts.

Moreover, we conducted the McNemar test with a Bonferroni correction on the responses to the question “Can you read the text?” for each anchor type with the three fonts and found no significant differences between any text pairs. Table R1 shows the participants’ answers to the question “Can you read the text?” In this question, one participant responded, “I cannot read the text” five times for these stimuli: “150” (easy-to-read fonts), “150” (hard-to-read fonts in gray), “25” (easy-to-read fonts), “25” (hard-to-read fonts in black), and “a very heavy person (kg)” (easy-to-read fonts). The participant might have misunderstood the intention of the experimental explanation given that he or she repeatedly answered that he or she could read the hard-to-read fonts but could not read the easy-to-read fonts. All the other participants were able to read all the text and understand the written content in all the fonts. These results indicated that the hard-to-read fonts (in black or gray) were perceived as difficult to read, but the written content could still be understood. Therefore, the results suggested that the stimuli used in Experiments 1 and 3 were suitable for use as anchors.

Furthermore, we performed multiple comparisons of the Wilcoxon signed-rank test with a Bonferroni correction on the response time for selecting the options (i.e., “I cannot read the text” or “I can read the text”) for each anchor type with the three fonts. We thought that we could measure the depth of cognitive processing of the stimuli by the response time. We excluded 13 responses that were more than 10 seconds. The Smirnov–Grubbs test showed that the responses were significant outliers ($p < 0.05$), and the other responses were not significant outliers. We found no significant differences between any pairs of fonts (see Figure R3). Although the easy-to-read fonts were easier to read than the hard-to-read fonts (in black or gray), we found no significant differences in the response times, possibly because the choice task was too easy for the participants.

To summarize the results, the hard-to-read fonts (in black or gray) were more difficult to read than the easy-to-read fonts. Moreover, the participants were able to read the texts and understand the written content in all stimuli conditions. Therefore, the stimuli were suitable to be used to generate the anchoring effect. However, we found no significant differences in the response times for selecting the options, so we could not confirm whether the differences in fonts caused any difference in the depth of cognitive processing of the texts.

5 Reference

Diemand-Yauman, C., Oppenheimer, D. M., and Vaughan, E. B. (2011). Fortune favors the bold (and the italicized): Effects of disfluency on educational outcomes. *Cognition* 118, 111–115. http://doi.org/10.1016/j.cognition.2010.09.012

TABLE R1 | Stimuli used in the supplementary experiment. "No" indicates that the participants answered, "I cannot read the text." "Yes" indicates that they answered, "I can read the text."

	No	Yes
150 [Easy-to-read fonts]	1	29
150 [Hard-to-read fonts in black]	0	30
150 [Hard-to-read fonts in gray]	1	29
25 [Easy-to-read fonts]	1	29
25 [Hard-to-read fonts in black]	1	29
25 [Hard-to-read fonts in gray]	0	30
A very heavy person (kg) [Easy-to-read fonts]	1	29
A very heavy person (kg) [Hard-to-read fonts in black]	0	30
A very heavy person (kg) [Hard-to-read fonts in gray]	0	30
A very heavy person 150 (kg) [Easy-to-read fonts]	0	30
A very heavy person 150 (kg) [Hard-to-read fonts in black]	0	30
A very heavy person 150 (kg) [Hard-to-read fonts in gray]	0	30
A very light person (kg) [Easy-to-read fonts]	0	30
A very light person (kg) [Hard-to-read fonts in black]	0	30
A very light person (kg) [Hard-to-read fonts in gray]	0	30
A very light person 25 (kg) [Easy-to-read fonts]	0	30
A very light person 25 (kg) [Hard-to-read fonts in black]	0	30
A very light person 25 (kg) [Hard-to-read fonts in gray]	0	30

a

150

b

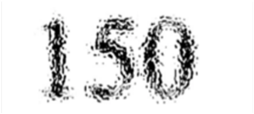

c

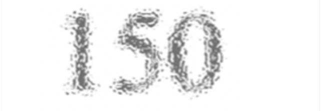

d

25

e

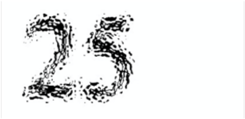

f

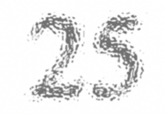

g

とても重い体重 (kg)

h

とても重い体重 (kg)

i

とても重い体重 (kg)

j

とても軽い体重 (kg)

k

とても軽い体重 (kg)

l

とても軽い体重 (kg)

m

とても重い体重 150 (kg)

n

とても重い体重 150 (kg)

o

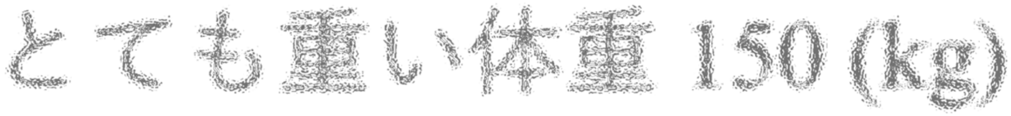

p

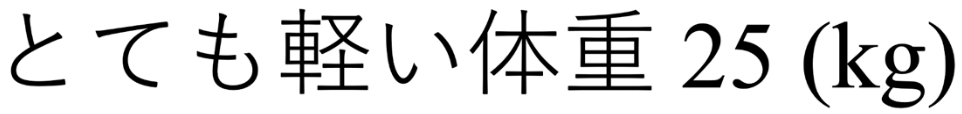

q

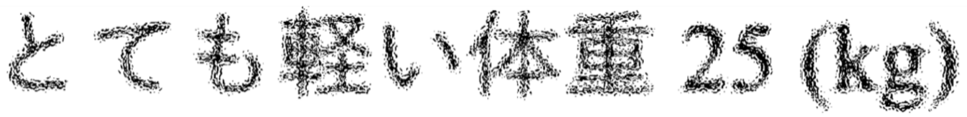

r

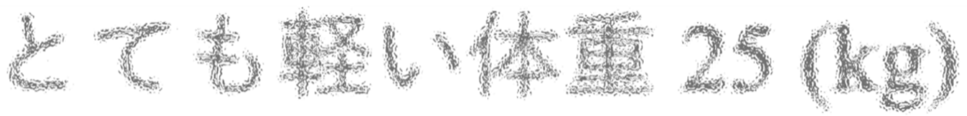

FIGURE R1 | R1 a, b, and c denote “150.” Figure R1 d, e, and f denote “25.” Figure R1 g, h, and i denote “a very heavy person (kg).” Figure R1 j, k, and l denote “a very light person (kg).” Figure R1 m, n, and o denote “a very heavy person 150 (kg).” Figure R1 p, q, and r denote “a very light person 25 (kg).” Figure R1 b, e, h, k, n, and q were presented as blurred texts with black colors in Experiment 1. Figure R1 c, f, i, l, o, and r were presented as blurred texts with gray colors in Experiment 3.

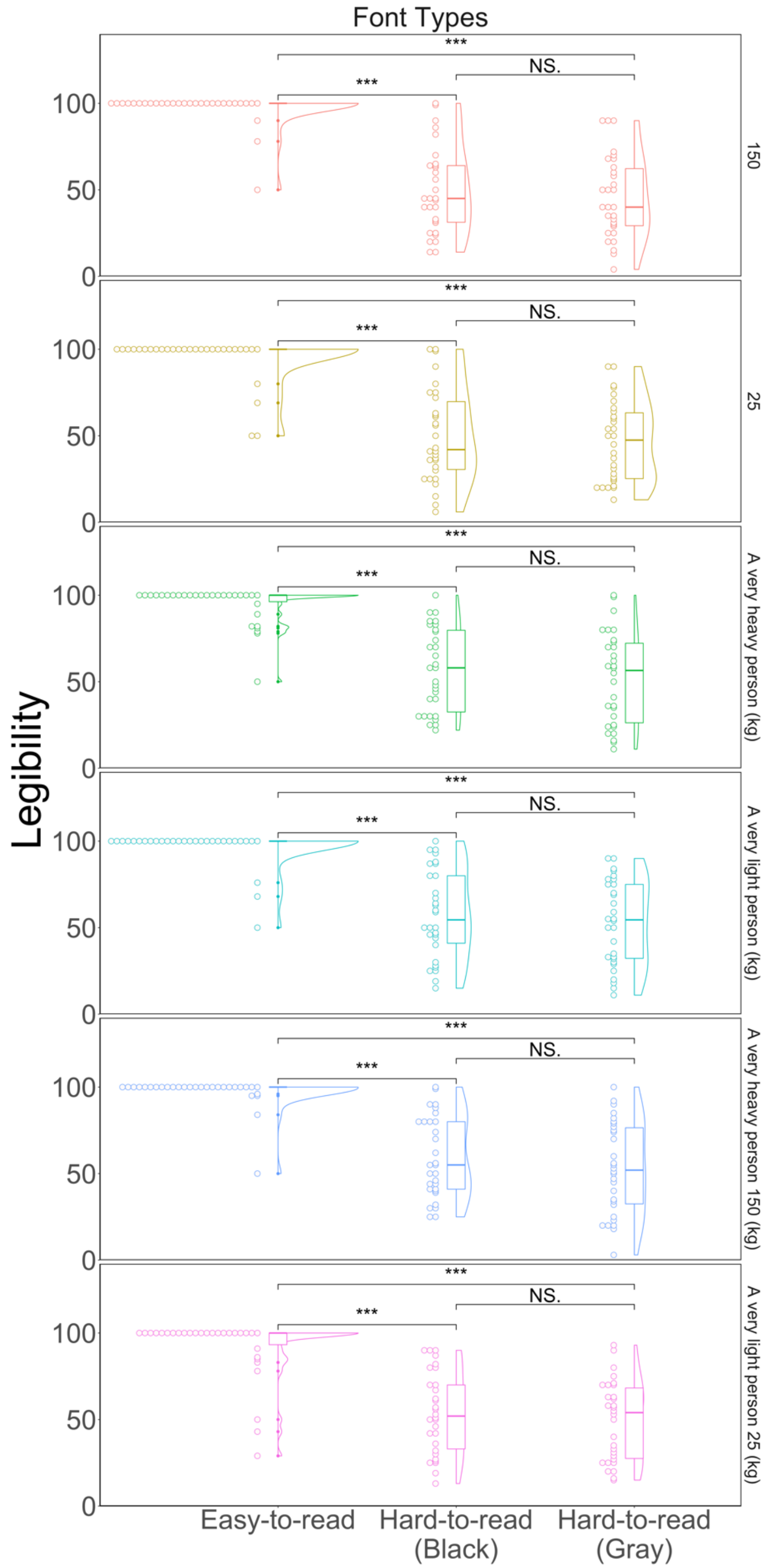

FIGURE R2 | Violin and dot plots of the participants' legibility ratings. ***$p < 0.001$, *NS* $p > 0.05$.

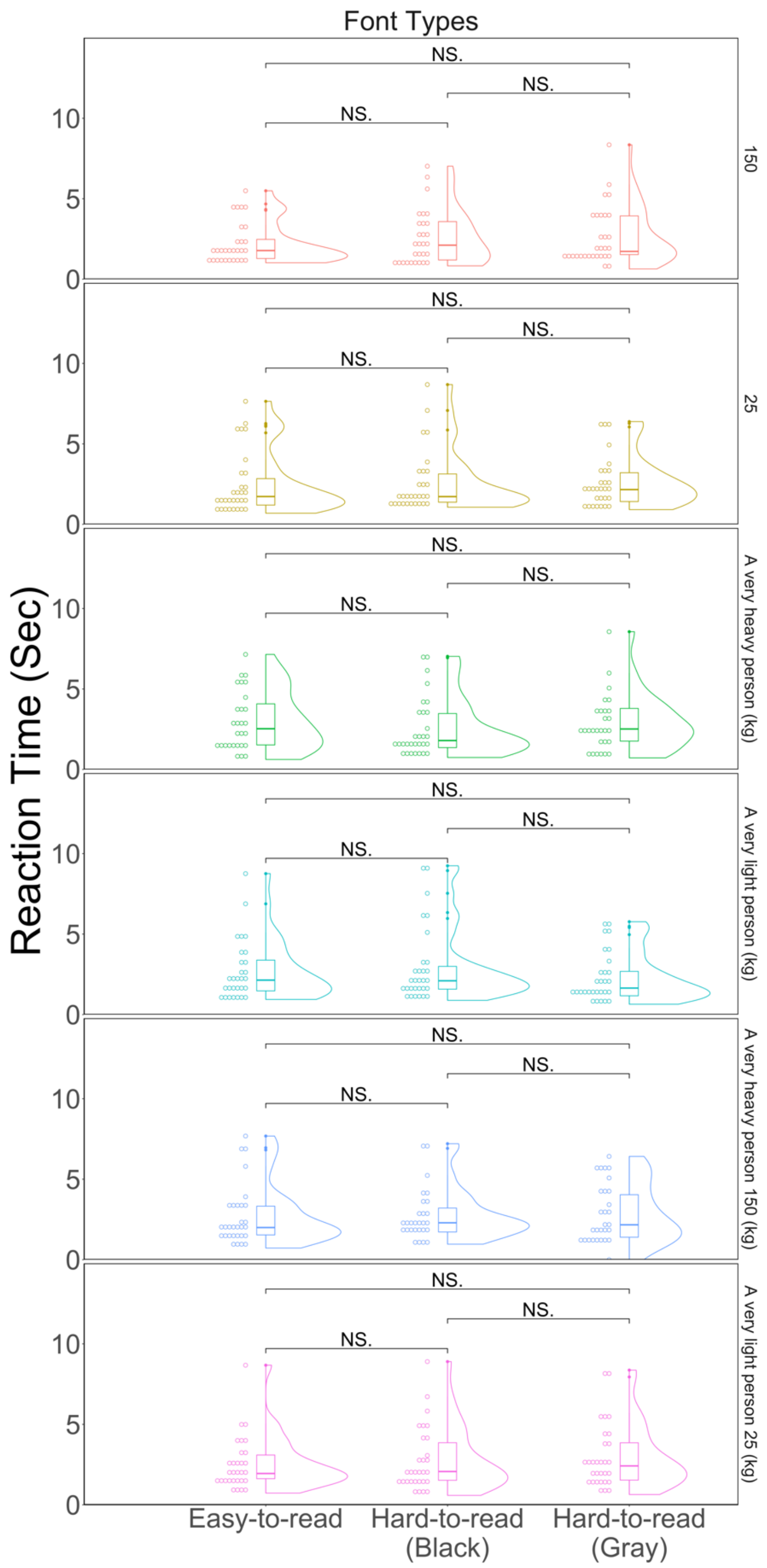

FIGURE R3 | Violin and dot plots of the participants' option response times. *NS* $p > 0.05$.
